# The Severity of Acute Kidney and Lung Injuries Induced by Cecal Ligation and Puncture Is Attenuated by Menthol: Role of Proliferating Cell Nuclear Antigen and Apoptotic Markers

**DOI:** 10.3389/fmed.2022.904286

**Published:** 2022-06-23

**Authors:** Aliaa Anter, Al-Shaimaa F. Ahmed, Asmaa S. A. Hammad, Waleed Hassan Almalki, Sara Mohamed Naguib Abdel Hafez, AlShaimaa W. Kasem, Mohamed A. El-Moselhy, Mohammad W. Alrabia, Ahmed R. N. Ibrahim, Mahmoud El-Daly

**Affiliations:** ^1^Department of Pharmacology and Toxicology, Faculty of Pharmacy, Minia University, Minya, Egypt; ^2^Department of Pharmacology and Toxicology, Umm Al-Qura University, Makkah, Saudi Arabia; ^3^Department of Histology and Cell Biology, Faculty of Medicine, Minia University, Minya, Egypt; ^4^Department of Pathology, Faculty of Medicine, Minia University, Minya, Egypt; ^5^Department of Clinical Pharmacy and Pharmacology, Ibn Sina National College for Medical Studies, Jeddah, Saudi Arabia; ^6^Department of Microbiology and Medical Parasitology, Faculty of Medicine, King Abdulaziz University, Jeddah, Saudi Arabia; ^7^Department of Clinical Pharmacy, College of Pharmacy, King Khalid University, Abha, Saudi Arabia; ^8^Department of Biochemistry, Faculty of Pharmacy, Minia University, Minya, Egypt

**Keywords:** menthol, cecal ligation and puncture, AKI, ALI, PCNA

## Abstract

**Objective:**

Sepsis-induced acute lung injury (ALI) and acute kidney injury (AKI) are major causes of mortality. Menthol is a natural compound that has anti-inflammatory and antioxidative actions. Since exaggerated inflammatory and oxidative stress are characteristics of sepsis, the aim of this study was to evaluate the effect of menthol against sepsis-induced mortality, ALI, and AKI.

**Methods:**

The cecal ligation and puncture (CLP) procedure was employed as a model of sepsis. Rats were grouped into sham, sham-Menthol, CLP, and CLP-Menthol (100 mg/kg, p.o).

**Key Findings:**

A survival study showed that menthol enhanced the survival after sepsis from 0% in septic group to 30%. Septic rats developed histological evidence of ALI and AKI. Menthol markedly suppressed sepsis induced elevation of tissue TNF-a, ameliorated sepsis-induced cleavage of caspase-3 and restored the antiapoptotic marker Bcl2.

**Significance:**

We introduced a role of the proliferating cell nuclear antigen (PCNA) in these tissues with a possible link to the damage induced by sepsis. PCNA level was markedly reduced in septic animals and menthol ameliorated this effect. Our data provide novel evidence that menthol protects against organ damage and decreases mortality in experimental sepsis.

## Introduction

Sepsis is a complex and serious complication in postoperative critical care patients, mainly because of infection. It is a major cause of ICU patient morbidity as well as mortality (1, 2). The condition is characterized by multiple organ damage, with the kidneys and the lungs being the most vulnerable organs (3). About 50% of patients with sepsis may experience acute kidney (AKI) and lung injuries (ALI). Despite all improved and labored treatment strategies to medicate sepsis patients, the mortality rate for patients with AKI and ALI is at alarming levels (up to 70%) (4, 5). Thus, the research aiming to identify novel therapies and prevention approaches, which should be both effective and safe, is a pressing need for sepsis management.

The underlying mechanisms of sepsis and its complications are not completely identified. During the acute phase of sepsis, microbial components trigger the immune and inflammatory cascades. However, the exaggerated release of cytokines during the acute stage of sepsis leads to multiple tissue damage. Sepsis-associated hemodynamic changes serve an initial function in counteracting foreign organisms tissue infiltration through inhibition of vascular tone and boosting the coagulation cascade (6, 7). This increased endothelial permeability during sepsis enhances infiltration of immune cells to the site of injury. This leads to intensive vascular leakage which insults the host tissues through excessive loss of intracellular plasma volume, subsequent hypotension, and decreased perfusion to vital organs. Thus, the host response becomes pathoadaptive and imposes a considerable threat to the host (8). Increased tissue infiltration by activated immune cells provokes tissue inflammation and excessive generation of reactive oxygen species (ROS), which are hallmarks of sepsis, and the associated multiple organ damage (7, 9). ROS-induced damage of the mitochondrial membrane and its altered permeability activates the mitochondria apoptotic cascade (10, 11). In sepsis, increased levels of TNF-α activate the external apoptotic pathways, which involves the activation of death receptors (12). Activated apical caspase-9 and -8, from either pathway, activate the effector caspases (caspases-3, -6, and -7). Intriguingly, activated caspase-3 mediates a feedback activation of caspase-8 and -9. Thus, besides its function as an effector caspase, caspase-3 maximizes the activation of apical caspases and the interaction between the two major apoptotic pathways (13). On the other hand, Bcl2 is an antiapoptotic protein, which maintains the permeability of the mitochondrial membrane and modulates cytochrome c release (14). Previous studies showed that the protein level of the proliferating cell nuclear antigen (PCNA) positively correlates with the regenerative capacity of tissues (15). PCNA interferes with apoptosis by binding procaspases, which prevents their activation and inhibits apoptosis (16).

Phytochemicals are promising therapeutic candidates for many disease conditions because of their antioxidant, anti-inflammatory, and immune system modulatory effects. Menthol, the main volatile ingredient of peppermint oil, has long been used in traditional medicine for its antispasmodic and carminative properties. Previous research highlighted the analgesic (17), antitussive (18), immune-modulatory (19), and the anti-apoptotic actions (20) of menthol. The anti-inflammatory and antioxidative (19–23) effects are of special importance to the current study. Besides, menthol is an ingredient of many pharmaceutical preparations effective in the management of respiratory diseases such as sinusitis, allergic rhinitis, and bronchitis (24). A recent study illustrated that menthol alleviated cigarette smoke-induced lung injury *via* suppression of oxidative stress and inflammation (25). Similarly, other studies reported menthol-induced liver and kidney protection against acute injury induced by various chemical insults (26, 27). Like the work by Liu et al. (25), the results of these studies showed that antioxidative and anti-inflammatory mechanisms are involved in menthol-induced protection (26, 27). In addition, menthol activates transient receptor potential (TRP) melastatin 8 (TRPM8) (28), which mediates its analgesic effects (29). On the other hand, pharmacological activation of TRPM8 was anti-inflammatory in a mouse model of colitis (30). The safety of menthol is well established, in an early study, oral administration of menthol up 667 mg/kg/day was not associated with any observed adverse effects (31). In another study, the plasma concentration of menthol was reported to reach 20 μM within 1 h in rats that have been administered 400 mg of menthol/kg body weight i.p (32). All menthol isomers are well absorbed after oral and are excreted as glucuronides. In rats, an extensive enterohepatic circulation is reported. For all isomers of menthol, a very low acute oral toxicity with LD_50_ values normally greater than 2,000 mg/kg bw has been reported (33).

Since exaggerated inflammatory and deregulated immune responses and increased oxidative stress are hallmarks of sepsis, we hypothesized that menthol would improve sepsis outcomes *via* its inflammatory and antioxidant properties. Thus, this study aimed to test the effect of menthol on the survival and acute organ injury in the kidney and lungs of experimentally induced septic animals. We evaluated the underlying mechanisms of such protective effects of menthol.

## Materials and Methods

### Animals and Experimental Design

Female Wistar rats, weighing between 250–300 g, were obtained from the animal care center of Nahda University at Beni Suef (NUB), Beni-Suef, Egypt. The rats were left for 1 week for acclimatization and kept at constant temperature and under a 12 h light-dark cycle with free access to food and water until the day of the experiment. This study was approved in January 2020 by The Commission on the Ethics of Scientific Research, Faculty of Pharmacy, Minia University (Approval Number: ES02/2020).

Sepsis was induced by cecal ligation and puncture procedure (CLP) as described previously (34). Briefly, animals were anesthetized by a mixture of ketamine (50 mg/kg) and xylazine (10 mg/kg), and a 2-cm ventral midline abdominal incision in the lower left quadrant of the body was performed. The cecum was exposed, ligated just below the ileocecal valve with a surgical 0.3-mm silk thread, and punctured twice with an 18-gauge needle. The punctured cecum was massaged gently to squeeze a small amount of the bowel content and placed back in the abdominal cavity, and the wounded abdominal wall was then sutured. Immediately after the operation, normal saline (3 mL/100 g) was injected subcutaneously. Sham-operated rats were subjected to all the previous steps except for the cecum ligation and puncture. The study groups were: Sham (vehicle-treated), Sham-treated with menthol (100 mg/kg, p.o) (35), CLP (vehicle-treated) and CLP-treated with menthol (100 mg/kg, p.o). Animals received either vehicle (distilled water) or menthol (as a suspension in distilled water) treatments 2 h after the CLP procedure. Menthol was obtained from Sigma-Aldrich Inc. (Cat. No. 63670). Since our study was the first to examine the effect of menthol on an animal model of sepsis, the dose, route and timing of administration were all based on several pilot studies on the effect of menthol on the survival of animals after CLP. The oral route of 100 mg/kg given to rats 2 h after CLP gave the best results in terms of survival. As previously stated in the introduction section, menthol administered by the oral route exhibits high bioavailability and safety (36).

For the survival study, separate groups were applied (*n* = 10/group) from the other study where the rats were observed for 10 days to report mortality. For the mechanistic study, a total of 28 rats were used, sham (*n* = 6), sham-menthol (*n* = 6), CLP (*n* = 10) and CLP-menthol (*n* = 6). Twenty-four hours after sepsis induction, all rats were sacrificed by exsanguination after an i.p injection of thiopental sodium (50 mg/kg).

### Assessment of Total Leukocytic Cell Counts and Total Protein in the Bronchoalveolar Lavage Fluid

The bronchoalveolar lavage fluid (BALF) was obtained by washing the airways three times with 0.5 ml of saline using a tracheal cannula (37). The BALF was used for the determination of total protein and WBC count. Briefly, after centrifugation (1,000 rpm, 10 min at 4°C) of the BALF, the cell pellet was resuspended in 0.5 ml PBS and the total cells were counted using a hemocytometer. Total protein was measured in the supernatant of the BALF based on its reaction with copper ions that produces a blue-violet color proportional to the concentration of the protein in the sample using a commercial kit (BioMed, Egypt).

### Assessment of Serum Creatinine

Blood was collected by exsanguination from thiopental-sedated animals and centrifuged after 10 min to obtain serum samples. Creatinine was determined according to the method described by **Schirmeister** (38) using a commercial kit from Biodiagnostic, Egypt.

### Histopathological Examination

Specimens from the left lung and kidney tissues were immediately cut into small pieces, fixed in 10% formol saline, and processed to get 5 μm thick paraffin sections for Hematoxylin and Eosin (H & E) study (39). Slides stained with H & E were microscopically analyzed by light microscopy (Olympus CX 23L). Blind pathological assessment was carried out on lung and kidney tissues from each group (*n* = 6). The lung injury was graded on a scale of 0–4 (0, absent; 1, light; 2, moderate; 3, strong; 4, intense) for alveolar wall thickness, intra-alveolar edema and congestion, and damaged membranes. The lung injury score was calculated as the mean of the scores for the separate parameters. Kidney injury was assessed for the percentages of glomerular damage, mesangial cells proliferation, tubular damage, tubular edema, and congestion that were scored as follows: 0 = none, 1 = 0–20%, 2 = 20–50%, 3 = 50–70%, 4 = more than 70%. For each animal, at least 10 fields from the lung or kidney sections were examined, and the average scores were recorded (40).

### Determination of Oxidative Stress Parameters in Lung and Kidney Tissue Homogenates

Tissue samples of the right lung and kidney were homogenized to assess the different biochemical markers. The content of the marker of lipid peroxidation malondialdehyde (MDA) was determined colorimetrically in lung and kidney tissue homogenates according to the method of Buege and Aust (41). The concentration of total nitrates was colorimetrically measured after reducing nitrates in the samples to nitrites by a cadmium reagent, and the ability of nitrite ions to form a colored azo-dye in the presence of the Griess reagents as previously described (42).

GSH concentration was measured colorimetrically according to the method described by Rifai (43). The activity of SOD was determined according to a previously described method (44) using a commercially available kit (Biodiagnostic, Egypt).

#### Immunohistochemical Assessment of Lung and Kidney Caspase-3, Bcl-2, Proliferating Cell Nuclear Antigen, and TNF-α

Paraffin-embedded sections of the lung and kidney specimens were processed for the immunohistochemical study (45). Immunohistochemical staining was carried out for cleaved caspase-3, B-cell lymphoma 2 (Bcl-2) protein, proliferating cell nuclear antigen (PCNA), and TNF-α using rabbit monoclonal anti-caspase-3 (catalog number ab32042, Abcam, Cambridge, United Kingdom), rabbit polyclonal anti-Bcl-2 (catalog number ab194583, Abcam, Cambridge, United Kingdom), mouse monoclonal anti-PCNA (catalog number ab29, Abcam, Cambridge, United Kingdom), and rabbit monoclonal anti-TNF-α (catalog number EPR21753-109, Abcam, Cambridge, United Kingdom), respectively. To ensure the specificity of the antibody reaction, a control experiment was performed following the same steps, but without the addition of the primary antibody. Positive controls samples were human vascular endothelial cells for caspase-3, human lung cancer for Bcl-2, liver tissue for PCNA, and rat splenocytes for TNF-α.

### Image Capture

The photography was performed in the Histology and Cell Biology Department, Faculty of Medicine, Minia University. An Olympus (U.TV0.5XC-3) light microscope with a digital camera was used in this experiment. Photomicrographs were assessed by Adobe Photoshop.

### Morphometric Study

Ten random non-overlapping fields for each slide from each group from lung and kidney tissues were selected and imaged using power X400 (46, 47). The mean surface area fraction of anti-cleaved caspase-3, PCNA, Bcl2, and TNF-α immuno-positive cells were measured using ImageJ software (version 1.51k, Wayne Rasband, National Institutes of Health, United States) for image analysis.

#### Statistical Analysis

The GraphPad Prism 6 (version 6.0; San Diego, CA, United States) software was used for statistical analyses. Data are expressed as mean ± SEM. The difference between means of different groups was analyzed using one-way analysis of variance (ANOVA), followed by the Tukey-Kramer post-ANOVA test for multiple comparisons. Animal survival data were analyzed using the Log-rank Mantel-Cox test. The results were considered statistically significant if the *p*-values were < 0.05. The correlation between parameters related to kidney injury or lung injury was determined by calculation of the Pearson correlation coefficient, r. The correlation is weak when *r* < | 0.3|, moderate when | 0.3| ≤ *r* ≤ | 0.7|. The correlation is considered strong when *r* > | 0.7|.

## Results

### Effect of Menthol on Sepsis-Induced Mortality

**FIGURE 1:**
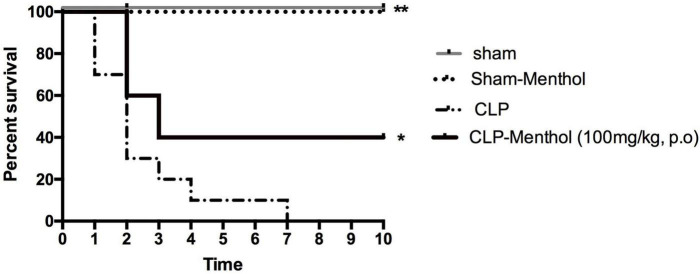
Effect of Menthol treatment on sepsis-induced mortality. Surgical induction of sepsis by the cecal ligation and puncture (CLP) procedure resulted in 100% mortality within 7 days (*n* = 10). Menthol treatment (100 mg/kg, p.o), 2 h after CLP, significantly improved the survival of septic rats (*n* = 10). Sham or sham menthol-treated rats (*n* = 10) showed no mortality. Survival analysis was carried out using the Log-rank Mantel-Cox test. * and ** Significantly different from the vehicle-treated CLP group at *p* < 0.05 and *p* < 0.01, respectively.

Rats in different groups (sham, sham-Menthol, CLP, or CLP-Menthol) were monitored every 24 h for 10 days after induction of sepsis. Figure 1 shows the survival rate of different groups. All rats in the CLP group died within 7 days after the surgical induction of sepsis. In contrast, Menthol (100 mg/kg) treatment reduced the overall mortality by 40% (*p* < 0.05). In addition, all septic animals in the menthol-treated group survived the first 24 h after surgery, while the vehicle-treated CLP rats showed 30% mortality. After 2 days of sepsis induction, the vehicle-treated CLP group showed higher mortality (70%) than the menthol-treated septic rats (40%). Three days after surgery, the menthol-treated group showed no more mortality (40% survival). Both the sham-operated or the sham-operated menthol-treated groups did not show any mortality. These results show the protective role of menthol treatment against mortality in septic rats.

### Protective Effect of Menthol on Sepsis-Induced Acute Lung Injury

**FIGURE 2:**
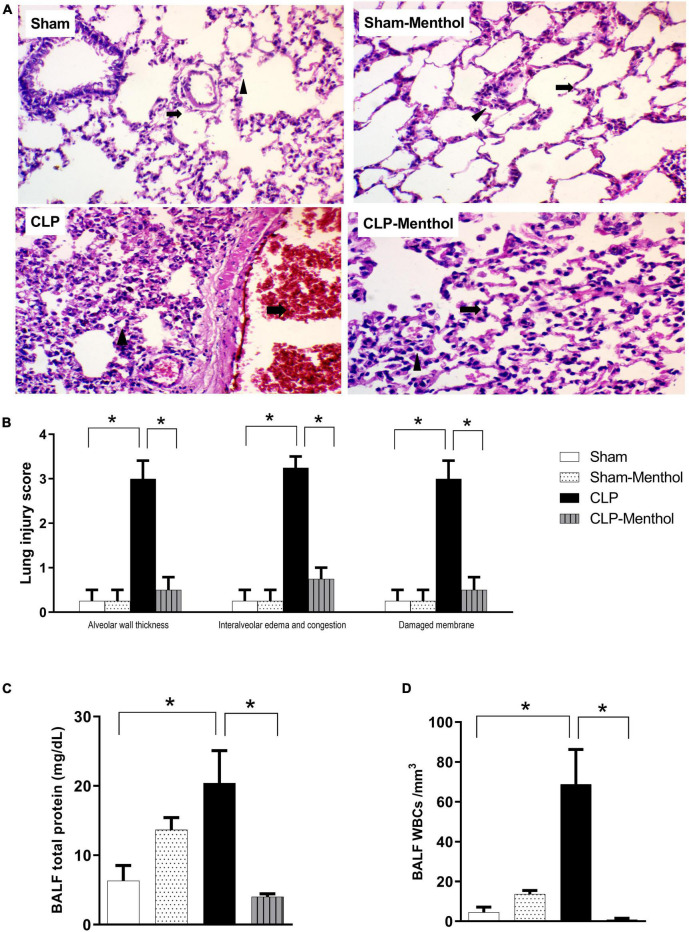
Effect of menthol on sepsis-induced acute lung injury. Sepsis was induced by CLP under general anesthesia as described under the “Materials and Methods” section. Animals were killed after 24 h of CLP and the lung injury was assessed by histological examination of H&E-stained (200×) sections **(A,B)** and by determination of the protein and total WBCs in the bronchoalveolar lavage (BALF; **C,D**) in all groups (*n* = 6 per group). **(A)** Representative photomicrographs showing that menthol treatment attenuated the histopathological changes induced by sepsis in the lung tissue. The sham groups (Sham and Sham-Menthol) showed normal lung alveolar spaces (arrow) separated by non-congested vascular spaces (arrowhead). The lung tissue of the vehicle-treated CLP group (CLP) showed thickened alveolar spaces with alveolar wall damage (arrow) separated by dilated and congested vascular spaces (arrowhead). Lung tissues from the menthol-treated septic rats (CLP-Menthol) showed normal alveolar spaces with intact alveolar wall (arrow) separated by mild congested vascular spaces (arrowhead). Summary of the mean values of the combined lung injury scores (at least 10 fields per section from 6 animals) of different groups is shown **(B)**. The total protein content **(C)** and the total leucocyte count **(D)** in BALF of different groups (*n* = 6) are also shown. Data represent the mean ± SEM of 6 independent observations. Data were analyzed by one-way ANOVA followed by the Tukey-Kramer post- test for multiple comparisons. *Significantly different at *p* < 0.05 compared to the CLP group.

We assessed the protective effect of menthol on lung tissues after induction of sepsis by histopathological examination of paraffin-fixed sections, and by determination of the total protein concentration and WBC count in BALF. H&E-stained lung sections from the sham groups showed normal alveolar spaces **(arrow)** separated by non-congested vascular spaces **(arrowhead)** as shown in Figure 2A. The histological examination of vehicle-treated CLP lung tissues revealed thickened alveolar spaces with alveolar wall damage **(arrow)** separated by dilated and congested vascular spaces (**arrowhead).** Sections obtained from the menthol-treated CLP group showed normal alveolar spaces with intact alveolar walls **(arrow)** separated by mild congested vascular spaces (**arrowhead).** The lung injury scores increased markedly in the CLP group in comparison with the menthol-treated CLP rats, which showed significantly decreased lung injury scores (Figure 2B).

Moreover, the total protein content and the total number of WBCs were significantly higher in the BALF collected from septic rats if compared with sham groups, while the treatment of CLP animals with menthol resulted a significant (*p* < 0.05) reduction of both total protein and the count of blood cells in collected BALF (Figures 2C,D).

### Protective Effect of Menthol on Sepsis-Induced Acute Kidney Injury

**FIGURE 3:**
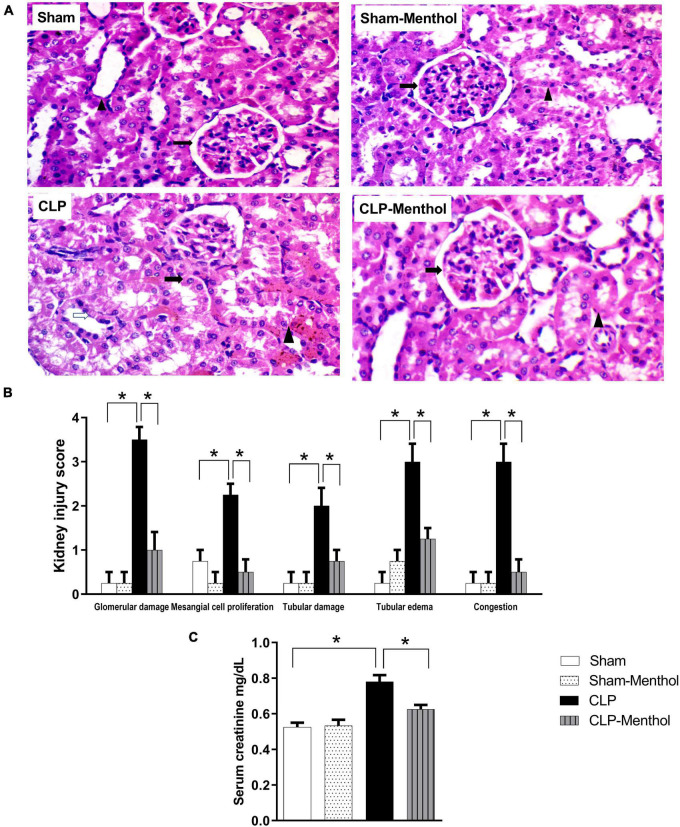
Effect of menthol treatment on sepsis-induced acute kidney injury. Sepsis was induced by CLP under general anesthesia as described under the “Materials and Methods” section. Animals were killed after 24 h of CLP and the kidney injury was assessed by histological examination of H&E-stained (200X) 5 μm sections **(A,B)** and by determination of serum creatinine concentration **(C)** in all groups (*n* = 6 per group). **(A)** Representative photomicrographs showing that menthol treatment attenuated the histopathological changes induced by sepsis in kidney tissues (H&E stain, 200X). The sham groups (Sham and Sham-Menthol) showed normal histological appearance with normal interstitial tissue without congestion or inflammatory cell infiltration. The glomeruli of the vehicle-treated CLP group (CLP) showed moderate damage and obliterated Bowman’s spaces. These sections showed proliferated mesangial cells (arrow) surrounded by tubules with focal areas of tubular damage (white arrow), tubular edema, compressed intratubular vascular spaces (arrowhead) and congested interstitial tissue. Menthol treated group showed renal glomerulus with normal histological appearance (arrow) surrounded by tubules (arrowhead) with normal interstitial tissue without congestion or inflammatory infiltrates. **(B)** Bar charts showing analysis of the mean values of combined kidney injury scores (at least 10 fields per section from 6 animals per group). **(C)** Bar graph showing the serum creatinine data from the different groups. All data represent the mean ± SEM of 6 independent observations. Data were analyzed by one-way ANOVA followed by the Tukey-Kramer post- test for multiple comparisons. *Significantly different at *p* < 0.05 compared to the CLP group.

The kidney tissues from sham or sham-menthol-treated groups showed normal histological appearance, normal interstitial tissue, and lack of congestion or inflammatory infiltration upon histological examination (Figure 3A). The CLP group showed moderately damaged renal glomeruli, obliterated Bowman’s spaces, proliferated mesangial cells (**arrow**) surrounded by tubules with focal areas of tubular damage **(white arrow),** tubular edema with compressed intratubular vascular spaces (**arrowhead**), and congested interstitial tissues. On the other hand, sections from the menthol-treated CLP group showed normal renal glomeruli (**arrow**) surrounded by tubules (**arrowhead**) with normal interstitial tissue, without congestion or inflammatory infiltrates (Figure 3A). Kidney injury scores were obviously increased in the vehicle-treated CLP group, while administration of menthol in CLP rats markedly reduced these scores (Figure 3B).

To evaluate kidney function, the serum creatinine level was measured in all groups. Induction of sepsis by the CLP procedure significantly (*p* < 0.05) increased the serum creatinine level compared with the sham group, while menthol treatment after CLP surgery significantly (*p* < 0.05) abolished this increase (Figure 3C).

### Menthol Reduces Oxidative and Improves Antioxidant Activity in Lung and Kidney Tissues of Septic Animals

**FIGURE 4:**
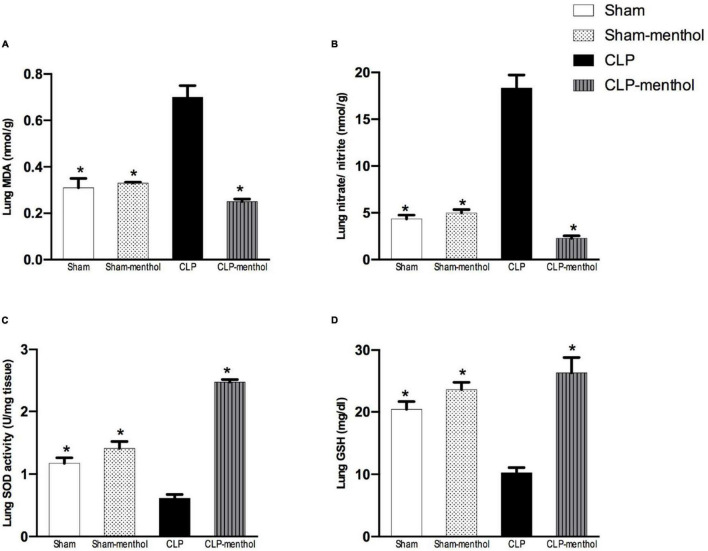
Menthol treatment improves pulmonary oxidative stress status and inhibits the sepsis-induced reduction of the antioxidant capacity of lung tissue. Menthol treatment of CLP rats ameliorated CLP-induced oxidative imbalance in lung tissues as shown with the results of **(A)**, malondialdehyde (MDA), **(B)** nitrate/nitrite, **(C)** activity of superoxide dismutase (SOD) and **(D)**, reduced glutathione (GSH). Menthol (100 mg/kg, p.o), 2 h after surgery. *n* = 6 per group. Data were analyzed by one-way ANOVA followed by Tukey’s post-test for multiple comparison. Data represent the mean ± SEM of 6 independent observations. *Significantly different from the CLP group at *p* < 0.05.

**FIGURE 5:**
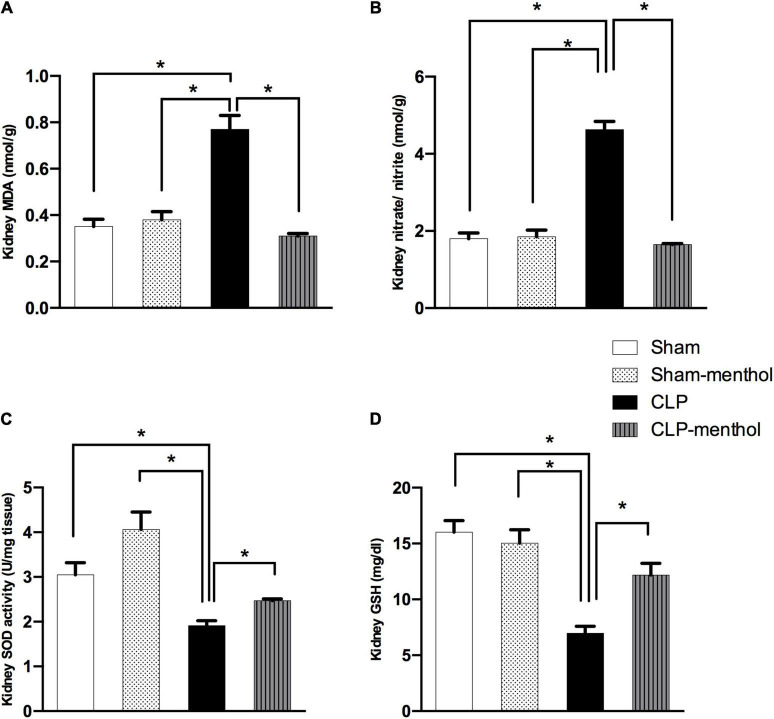
Menthol treatment attenuates renal oxidative stress and restores the antioxidant capacity of renal tissue. Cecal ligation and puncture enhanced oxidative stress which is normalized by menthol (100 mg/kg, p.o), 2 h after surgery as shown with the results of **(A)**, malondialdehyde (MDA), **(B)** nitrate/nitrite, **(C)** activity of superoxide dismutase (SOD) and **(D)**, reduced glutathione (GSH). *n* = 6 per group. Data were analyzed by one-way ANOVA followed by Tukey-Kramer post-test for multiple comparison. Data represent the mean ± SEM of 6 independent observations. *Significantly different from the CLP group at *p* < 0.05.

To evaluate the role of menthol on sepsis-induced increased oxidative stress in the lung and renal tissues, we measured the levels of lipid peroxidation, total nitrate/nitrite, SOD, and GSH (Figures 4, 5). The results revealed that induction of sepsis in the CLP model elevated MDA and total nitrate/nitrite levels in both lung and kidney homogenates. Sepsis induction significantly decreased the levels of the measured antioxidant markers; SOD and GSH. Administration of menthol 2 h after CLP induction improved the antioxidant capacity in comparison with the vehicle-treated CLP group. The tissues from the CLP-menthol group manifested significant reductions in MDA and total nitrate/nitrite levels in the lung and kidney homogenates, besides the restoration of sepsis-induced impairment of the tissue antioxidant capacity.

### Menthol Reduces Sepsis-Induced TNF-α Expression in Lung and Kidney Tissues

**FIGURE 6:**
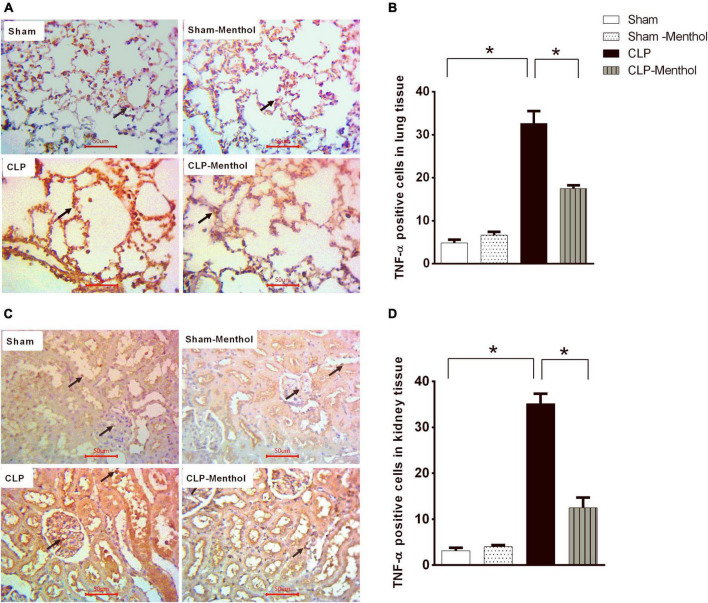
Effect of Menthol on TNF-α expression in the lung and kidney tissue of septic rats. Immunohistochemical staining of TNF-α in paraffin-embedded lung **(A)** and kidney **(C)** sections was carried out as described under the “Materials and Methods” section. Rabbit monoclonal anti-TNF-α (cat. # EPR21753-109, Abcam, Cambridge, United Kingdom) was used to probe tissue TNF-α. Ten random fields per slide from each group (*n* = 6) were selected and imaged (400X). The mean surface area fraction of TNF-α immuno-positive cells was measured using ImageJ software. Representative photomicrographs **(A)** showing TNF-α immunoreactivity in lung tissues. Bar charts **(B)** show semi-quantitative analysis of data in A from sections of the sham, sham-Menthol, CLP, and CLP-Menthol groups. TNF-α immunoreactivity in kidney sections **(C)** from all groups are semi-quantitatively analyzed in **(D)**. Data represent the mean ± SEM of 6 independent observations. Data were analyzed by one-way ANOVA followed by the Tukey-Kramer post-test for multiple comparisons. *Significantly different at *p* < 0.05 compared to the CLP group.

Both lung and renal tissues in the sham groups (Figures 6A,C) showed weak TNF-α immunostaining within the alveoli of the lung tissue, the renal glomeruli, or tubules. The vehicle-treated CLP group exhibited a significant (*p* < 0.05) increase in both the number and immune intensity of immunoreactive cells within the lining cells of the alveoli or glomerular or tubular cells if compared with the sham groups. Additionally, as shown in Figures 6B,D, menthol treatment after CLP significantly attenuated the increase in the number of TNF-α immunoreactive cells in the previously mentioned areas compared to the CLP group.

### Menthol Attenuates Sepsis-Induced Apoptosis in Lung and Kidney Tissues

**FIGURE 7:**
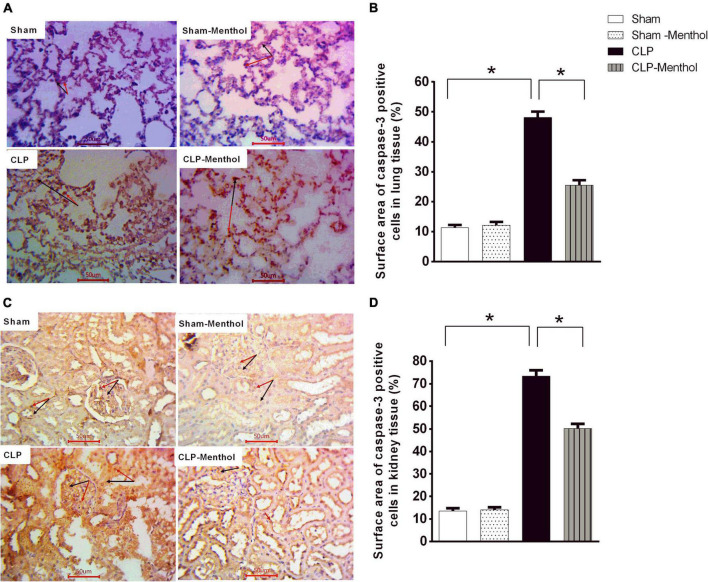
Caspase-3 expression in the lung and kidney tissues of septic rats. Tissue levels of cleaved caspase-3 were determined semi-quantitatively by immunohistochemical staining of paraffin-embedded lung **(A)** and kidney **(C)** sections (see “Materials and Methods” section). Rabbit monoclonal anti-caspase-3 (cat. # ab32042, Abcam, Cambridge, United Kingdom) was used. Ten random fields per slide from each group (*n* = 6) were selected and imaged (400×). The mean surface area fraction of caspase-3 immuno-positive cells was measured using ImageJ software. Representative photomicrographs **(A)** showing caspase-3 immunoreactivity in lung tissues. Summary data in the Bar charts **(B)** show semi-quantitative analysis of data in **(A)** from tissue sections of the sham, sham-Menthol, CLP, and CLP-Menthol groups. Data of caspase-3 immunoreactivity in kidney sections **(C)** from sham, sham-Menthol, CLP, and CLP-Menthol groups are semi-quantitatively analyzed in **(D)**. Data in the bar charts represent the mean ± SEM of 6 independent observations. Data were analyzed by one-way ANOVA followed by the Tukey-Kramer post- test for multiple comparisons. *Significantly different at *p* < 0.05 compared to the CLP group.

This study determined the immunoreactivity of the lung and kidney tissues to caspase-3 as a marker of cellular apoptosis. Data in Figures 7A,C show that both lung and renal tissues in the sham groups (Sham and Sham-Menthol) showed faint caspase-3 immunostaining; either cytoplasmic and/or nuclear, within the cells lining the alveoli of lung tissue, in renal glomerular, or tubular cells. Samples from septic rats exhibited a clear increase in the number and intensity of immunoreactive cells within these cells; either cytoplasmic and/or nuclear, if compared to the sham group. The sections of the menthol-treated CLP group revealed a significant (*p* < 0.05) decrease in the number of immunoreactive cells compared to the vehicle-treated CLP group (Figures 7B,D).

**FIGURE 8:**
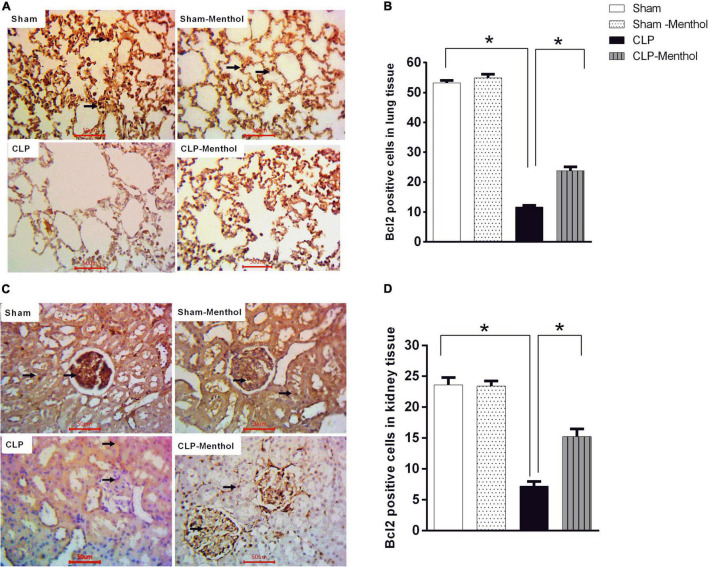
Bcl-2 expression in the lung and kidney tissues in different groups. Protein expression of Bcl-2 was achieved by immunohistochemical staining of paraffin-embedded lung **(A)** and kidney **(C)** sections was carried out after probing the sections with rabbit polyclonal anti- Bcl-2 (cat. # ab194583, Abcam, Cambridge, United Kingdom). The mean surface area fraction of Bcl-2 immuno-positive cells form images (400X) was measured using ImageJ software. Representative photomicrographs **(A)** showing the effect of menthol treatment on Bcl-2 immunoreactivity in lung tissues. A semi-quantitative analysis of the immunostaining data from ten random fields per slide from each group (*n* = 6) is depicted in **(B)**. Data of Bcl-2 immunoreactivity in the kidney sections from different groups are semi-quantitatively analyzed in **(D)**. Data represent the mean ± SEM of 6 independent observations. Data were analyzed by one-way ANOVA followed by the Tukey-Kramer post- test for multiple comparisons. *Significantly different at *p* < 0.05 compared to the CLP group.

We also examined the expression level of the Bcl2 protein, which negatively regulates cellular apoptosis, by immunohistochemistry. In contrast with caspase-3 results, the sham groups showed strong Bcl2 nuclear immunostaining within the lining cells of the alveoli of lung tissue, in renal glomeruli, or tubules (Figures 8A,C). In the CLP model, lung and kidney tissues exhibited a significant (*p* < 0.05) decrease in Bcl2-immunoreactive cells within the lining cells of the alveoli, the glomeruli, or tubular cells, if compared with the sham groups. Treatment of septic rats with menthol led to a significant (*p* < 0.05) increase in Bcl2-immunoreactivity compared with the CLP group (Figures 8B,D).

### Menthol Restores Proliferating Cell Nuclear Antigen Expression in the Lung and Kidney Tissues of Septic Rats

**FIGURE 9:**
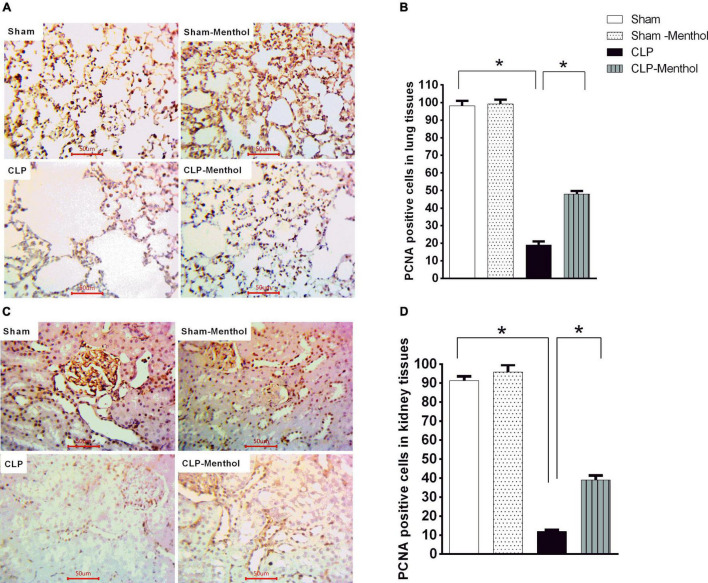
Menthol attenuates sepsis-induced reduction of PCNA expression in lung and kidney tissues. Determination of the tissue levels of PCNA was carried out by immunohistochemical staining of paraffin-embedded lung **(A)** and kidney **(C)** sections, as described in the “Materials and Methods” section. Mouse monoclonal anti-PCNA (cat. # ab29, Abcam, Cambridge, United Kingdom) was used in this experiment. Images of 10 random fields per slide from each group (*n* = 6) were selected and the mean surface area fraction of PCNA immuno-positive cells was measured using ImageJ software. Representative photomicrographs **(A)** showing PCNA immunoreactivity in lung tissues, and the Bar charts **(B)** show a semi-quantitative analysis of the data from all groups. Photomicrographs showing PCNA immunoreactivity in the kidney sections are shown **(C)**, which are semi-quantitatively analyzed in **(D)**. Data in the bar charts represent the mean ± SEM of 6 independent observations. Data were analyzed by one-way ANOVA followed by the Tukey-Kramer post- test for multiple comparisons. *Significantly different at *p* < 0.05 compared to the CLP group.

The proliferating cell nuclear antigen (PCNA) has an important role in nucleic acid metabolism during replication and repair processes. It is also involved in cell proliferation after injury and is an endogenous inhibitor of cell apoptosis. The results of this study showed that lung and kidney tissues from the sham groups (sham and sham-menthol) showed strong nuclear PCNA signal within the lining cells of the alveoli, the renal glomeruli, and tubules (Figures 9A,C). Lung and kidney tissues from the vehicle-treated septic rats showed an obvious decrease in nuclear PCNA immune-intensity within these cells, when compared to the sham groups. Interestingly, lung and kidney sections from the CLP-menthol group showed significantly elevated nuclear PCNA immunoreactivity compared with the CLP group (Figures 9B,D).

### Correlation Analysis Between the Measured Parameters and Kidney or Lung Injury

**FIGURE 10:**
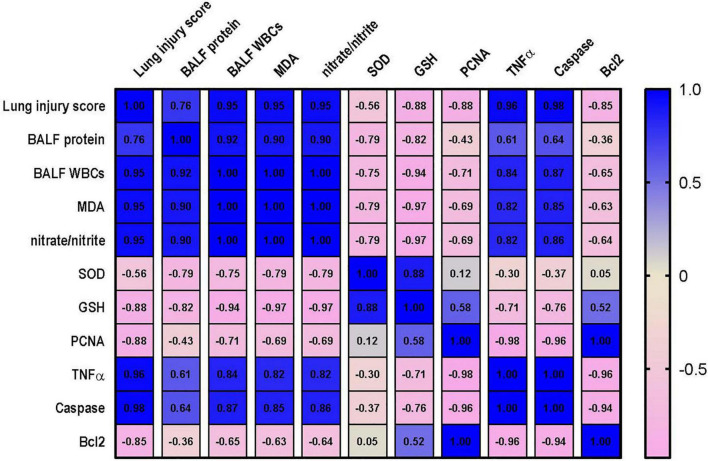
Correlation analysis of lung injury and measured parameters. Correlation matrix between lung injury score and related parameters. Data from different experiments were analyzed for correlation using the Pearson correlation coefficient (r). Positive values indicate a positive correlation while negative values indicate a negative one. When r is in the range of | 0.3| –| 0.7|, this indicates a moderate correlation, and when *r* > | 0.7|, this means a strong correlation. BALF, bronchoalveolar lavage fluid; MDA, malondialdehyde; SOD, superoxide dismutase; GSH, reduced glutathione; PCNA, proliferating cell nuclear antigen; TNF–α, tumor necrosis factor α; Bcl2, B cell lymphoma 2.

**FIGURE 11:**
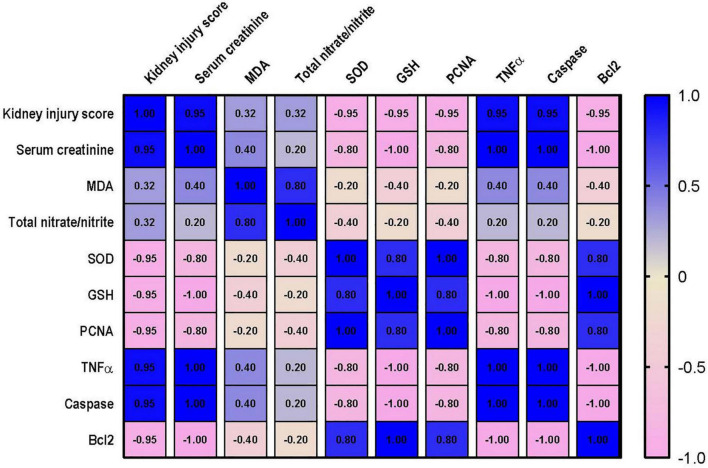
Correlation analysis of Kidney injury and measured parameters. Correlation matrix between kidney injury score and related parameters. Data from different experiments were analyzed for correlation using the Pearson correlation coefficient (r). Positive values indicate a positive correlation while negative values indicate a negative one. When r is in the range of | 0.3| –| 0.7|, this indicates a moderate correlation, and when *r* > | 0.7|, this means a strong correlation. MDA, malondialdehyde; SOD, superoxide dismutase; GSH, reduced glutathione; PCNA, proliferating cell nuclear antigen; TNF–α, tumor necrosis factor α; Bcl2, B cell lymphoma 2.

Correlation analysis shows that lung injury score possesses a strong correlation with the levels of MDA, nitrate/nitrite, caspase and TNF-α and a strong negative correlation with level of Bcl2, PCNA and GSH. However, only moderate negative correlation was detected between lung injury score and SOD as shown in Figure 10. Data also reveal that kidney injury score has a strong positive correlation with the level of serum creatinine, TNF-α and caspase. In addition to a strong negative correlation with GSH, Bcl2 and PCNA (Figure 11). However, Kidney injury is moderately correlated with total nitrate/nitrite and MDA. Data are presented as correlation matrix of the Pearson’s correlation coefficient between the measured parameters.

## Discussion

Multiple organ damage during the pathogenesis of sepsis results from the aggressive activation of the inflammatory and oxidative stress cascades and contributes to the high mortality rate characteristic of sepsis (9). In the current study, induction of experimental sepsis in rats induced acute lung and kidney injury and significant mortality. Our results show, for the first time, that menthol administration, 2-h after induction of sepsis, effectively enhanced the survival of septic rats and alleviated sepsis-induced lung and kidney tissue damage.

In this study, we observed a decline of function in the lung and kidneys of septic rats. Higher serum creatinine of septic compared with non-septic animals and infiltration of protein and leukocytes in the BALF are indicators of kidney and lung injury, respectively, which correlates with the high mortality rate observed in the septic rats. The kidney and lungs of septic rats exhibited high levels of tissue damage upon histological examination, which further highlights the detrimental effects of sepsis on different organs. Interestingly, oral treatment with menthol (100 mg/Kg), 2 h after surgery, ameliorated the impaired kidney function and restored the level of proteins and leukocytes in the BALF of septic rats. Histopathological findings of the lung and kidney tissues supported the beneficial effects of menthol. Thus, menthol effectively domesticated the state of sepsis and successfully preserved organ functions. These ameliorative effects of menthol treatment improved the survival of the menthol-treated CLP group (40%) compared with the septic rats (0%) by the end of the 10-days observation period. Recent studies showed that menthol-containing herbal extracts protect the kidneys in chemotherapy- (48) and gentamicin-induced (49) nephrotoxicity. Others have shown that menthol inhibited pulmonary inflammation and epithelial remodeling when inhaled by asthmatic animals (50).

The exaggerated inflammatory response during sepsis triggers the massive formation of reactive oxygen species (ROS). The mitochondria act both as a significant source and as a target for ROS. Sepsis provokes a cellular energy crisis, with systemic activation of the mitochondrial tricarboxylic acid cycle, and gluconeogenesis (51, 52). Such metabolic changes augment the production of reactive oxygen species (ROS)—key contributors to the pathology of sepsis. Increased ROS generation alters the chemistry of cellular proteins through nitrosylation, oxidation, and acetylation, not to mention the deterioration of mitochondrial membrane function because of increased lipid peroxidation (53). The enzymes of the electron transport chain are highly sensitive to oxidative stress, which contributes to the uncoupling of oxidative phosphorylation, and increased ROS production (51, 54). Thus, ROS-induced alteration of mitochondrial membrane permeability and respiratory enzymes switches on a ROS circuit, where increased ROS levels induce further ROS release from the mitochondria (54, 55). As shown by our data, induction of sepsis by CLP markedly increased oxidative stress-related parameters and decreased the activity of the endogenous antioxidant system. Menthol treatment, however, restored the antioxidant activity in the lung and kidneys of septic rats, which conforms with its well-documented antioxidant effects (56–59). Sepsis reduces the activity of heat shock protein-70 (60), which is involved in a variety of biological activities, including the reduction of oxidative stress. Interestingly, the results of a previous study showed that menthol-treated rats exhibited higher HSP70 expression, which correlated to its mucosal protection (20), and may also explain its antioxidant effect reported in our study.

TNF-α-mediated signaling augments mitochondrial ROS generation (11). Septic conditions manifest high levels of TNF-α in clinical (61) and experimental (62) settings. Since our data showed that menthol reduced the expression of lung and kidney TNF-α, this finding provides another explanation for the observed reduction in ROS levels within these tissues. The inhibitory effect of menthol on tissue TNF-α expression might depend on its potential activation of TRPM8 channels, which may serve an anti-inflammatory function to balance the pro-inflammatory responses of other TRP channels, such as TRPV1 and TRPA1 (63). Since activation of TRPM8 by menthol occurs with a low EC_50_ 4–80 μM (64) compared to other targets [refer to a comprehensive review on menthol targets (33)], we can argue that the effects were are observing can be largely attributed to activation or TRMP8 channels.

Activation of TRPM8 by menthol significantly abolished the Angiotensin-II-evoked oxidative stress and hydrogen peroxide release in vascular smooth muscles cells (65). The antioxidant mechanism of menthol involved TRPM8-dependent antagonism of the angiotensin-triggered induction of NADPH oxidases NOX1 and NOX4 (65). When TRPM8 channels are activated, the macrophages express and release the anti-inflammatory cytokine interleukin 10 (IL-10), while the release of TNF-α decreases (66). The well-known cooling effect of menthol might explain its inhibition of TNF-α expression. The study by Wang et al. (67) demonstrated that both menthol and cold stress inhibit the release of TNF-α through interaction with NF-κB; the nuclear transcription factor controlling the production of TNF-α and other pro-inflammatory mediators (68).

In our study, rats subjected to CLP showed a significant elevation of both ROS and TNF-α, which justifies the increased expression of active caspase-3 within lung and kidney tissues and explains the observed functional deterioration. Meanwhile, menthol effectively mitigated apoptosis, which is clear from our data showing the reduction in the lung and kidney expression of caspase-3. In our study, menthol inhibited the sepsis-induced reduction of tissue Bcl2 levels, parallel to menthol-induced tissue protection and improved survival. The protective effects of Bcl2 against acute lung injury might explain the protective effects of menthol in the current work. A recent study showed that besides the antiapoptotic function of Bcl2, its overexpression inhibits mitophagy *via* modulating the PINK1/Parkin pathway in a model of acute lung injury (69). Moreover, the enhanced expression of Bcl2 induced by menthol in septic rats may explain its ability to improve the pulmonary epithelial barrier. Although further elucidation of this effect is required, the recent findings of Otani et al. (70) support it. Their results suggested that the upregulation of Bcl2 counteracts the dysregulated permeability of intestinal cells in a mouse CLP model. Further, other researchers (71) recently proposed a relation between blood Bcl2 levels and survival in septic patients. Thus, the modulatory effect of menthol, and possibly other TRPM8 agonists, on Bcl2 expression and function, and its effect on the prognosis of sepsis is intriguing and needs further investigation.

Consistent with our findings, experimental induction of sepsis reduced the expression of PCNA in hepatocytes (72), and cardiomyocytes (73). In the current work, menthol preserved PCNA levels in the lung and kidneys of septic rats, which is in harmony with the observed reduction of activated caspase-3 expression. Experimental induction of severe sepsis reduced the expression of PCNA, which correlated with diminished hepatic tissue regenerative capacity (74). These findings (74), and ours, are further supported by the results of Abcejo et al. (72). Their results showed that PCNA expression inversely correlates with disease severity and mortality. Animals with severe sepsis displayed lower PCNA levels and higher mortality compared with those having a moderate phenotype (72). The CLP procedure adopted in our study represents a severe sepsis phenotype and shows a mortality profile comparable to the severe sepsis presented in the study by Abcejo et al. (72), which explains the decreased expression of PCNA observed in our untreated septic rats. Thus, the ability of menthol to preserve renal and pulmonary levels of PCNA, although partial, might provide further explanation for the enhanced survival of menthol-treated septic rats. However, whether this preservation of PCNA level is a consequence of the menthol-induced reduction of sepsis severity *via* reducing oxidative stress and inflammation or is a direct effect of menthol is yet to be answered. Nonetheless, preservation of PCNA levels in the lung and kidney of menthol-treated rats apparently contributes to lower organ damage and improves survival after CLP.

There are several limitations in our study, including the inclusion of a standard drug, which is actually hard to find in sepsis research as there are no definite treatment of sepsis or its associated organ dysfunction. Also, our study would be strengthened by the measurement of plasma menthol concentration as well as its concentration in the kidneys or lungs. In addition, because menthol appears to be a molecule with multiple cellular targets, future studies using specific antagonists would help in identifying the actual mechanism of menthol action in sepsis.

## Conclusion

**FIGURE 12:**
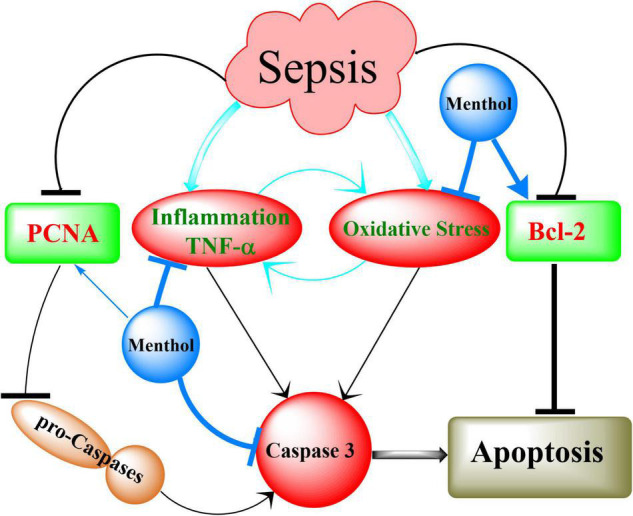
Summary of the mechanisms involved in menthol-induced protection against sepsis. Induction of sepsis by CLP upregulates inflammation (TNF-α) and oxidative stress, which in turn activates caspases to execute cellular apoptosis. This effect is augmented by destabilization of the antiapoptotic factor Bcl-2. Downregulation of proliferating cell nuclear antigen (PCNA) contributes to the conversion of procaspases into active caspases (Caspase 3). Menthol *via* inhibition of oxidative stress and inflammation on one hand, and preservation of PCNA and Bcl-2 of the other, abrogates sepsis-induced organ damage.

In conclusion, the results of our study showed for the first time the protective effects of oral menthol treatment against sepsis-induced mortality and damage in the lung and kidneys of septic rats. The protective mechanisms triggered by menthol include anti-oxidative, anti-inflammatory, and anti-apoptotic pathways (Figure 12). These findings open new horizons to discover novel targets for the management of sepsis.

## Data Availability Statement

The raw data supporting the conclusions of this article will be made available by the authors, without undue reservation.

## Ethics Statement

The animal study was reviewed and approved by the Commission on the Ethics of Scientific Research, Faculty of Pharmacy, Minia University (Approval Number: ES02/2020).

## Author Contributions

AAn, AAh, ME-D, and AH: execution of experiments, sample collection, and data handling and manuscript writing and revision. WA: design of the experiment, manuscript writing and revision, and funding source. WA and SA: histopathology and the immunohistochemistry studies and revision and approval of manuscript. ME-M and MA: critical manuscript revision, data analysis, and approval of final manuscript. AI: critical manuscript revision, data analysis, approval of final manuscript and funding. All authors listed have made a substantial, direct, and intellectual contribution to the work, and approved it for publication.

## Conflict of Interest

The authors declare that the research was conducted in the absence of any commercial or financial relationships that could be construed as a potential conflict of interest.

## Publisher’s Note

All claims expressed in this article are solely those of the authors and do not necessarily represent those of their affiliated organizations, or those of the publisher, the editors and the reviewers. Any product that may be evaluated in this article, or claim that may be made by its manufacturer, is not guaranteed or endorsed by the publisher.
