# Corrigendum: The severity of acute kidney and lung injuries induced by cecal ligation and puncture is attenuated by menthol: Role of proliferating cell nuclear antigen and apoptotic markers

**DOI:** 10.3389/fmed.2022.1024554

**Published:** 2022-09-22

**Authors:** Aliaa Anter, Al-Shaimaa F. Ahmed, Asmaa S. A. Hammad, Waleed Hassan Almalki, Sara Mohamed Naguib Abdel Hafez, AlShaimaa W. Kasem, Mohamed A. El-Moselhy, Mohammad W. Alrabia, Ahmed R. N. Ibrahim, Mahmoud El-Daly

**Affiliations:** ^1^Department of Pharmacology and Toxicology, Faculty of Pharmacy, Minia University, Minya, Egypt; ^2^Department of Pharmacology and Toxicology, Umm Al-Qura University, Makkah, Saudi Arabia; ^3^Department of Histology and Cell Biology, Faculty of Medicine, Minia University, Minya, Egypt; ^4^Department of Pathology, Faculty of Medicine, Minia University, Minya, Egypt; ^5^Department of Clinical Pharmacy and Pharmacology, Ibn Sina National College for Medical Studies, Jeddah, Saudi Arabia; ^6^Department of Microbiology and Medical Parasitology, Faculty of Medicine, King Abdulaziz University, Jeddah, Saudi Arabia; ^7^Department of Clinical Pharmacy, College of Pharmacy, King Khalid University, Abha, Saudi Arabia; ^8^Department of Biochemistry, Faculty of Pharmacy, Minia University, Minya, Egypt

**Keywords:** menthol, cecal ligation and puncture, AKI, ALI, PCNA

In the published article, there was an error in Figure 4 as published. The figure was a duplicate of Figure 5. The corrected Figure 4 and its caption appear below.

**Figure 4:**
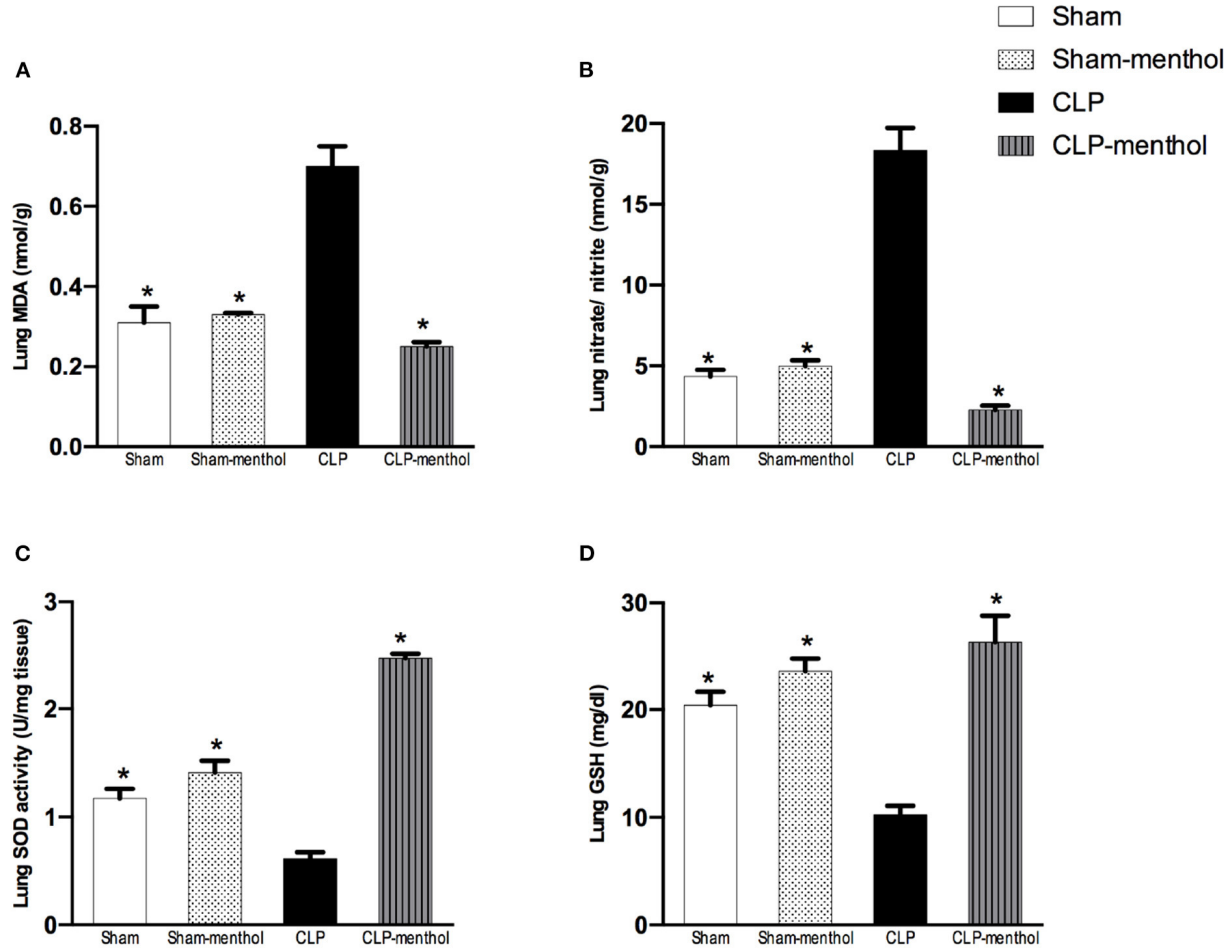
Menthol treatment improves pulmonary oxidative stress status and inhibits the sepsis-induced reduction of the antioxidant capacity of lung tissue. Menthol treatment of CLP rats ameliorated CLP-induced oxidative imbalance in lung tissues as shown with the results of **(A)**, malondialdehyde (MDA), **(B)** nitrate/nitrite, **(C)** activity of superoxide dismutase (SOD) and **(D)**, reduced glutathione (GSH). Menthol (100 mg/kg, p.o), 2 h after surgery. *n* = 6 per group. Data were analyzed by one-way ANOVA followed by Tukey's post-test for multiple comparison. Data represent the mean ± SEM of 6 independent observations. ^*^Significantly different from the CLP group at *p* < 0.05.

The authors apologize for this error and state that this does not change the scientific conclusions of the article in any way. The original article has been updated.

## Publisher's note

All claims expressed in this article are solely those of the authors and do not necessarily represent those of their affiliated organizations, or those of the publisher, the editors and the reviewers. Any product that may be evaluated in this article, or claim that may be made by its manufacturer, is not guaranteed or endorsed by the publisher.

